# Efficacy and safety of cannabidiol (CBD) on reducing pain and functional impairment associated with exercise-induced muscle injury: a randomized placebo-controlled feasibility trial

**DOI:** 10.1186/s42238-026-00431-x

**Published:** 2026-03-31

**Authors:** JW Stauffer, JA Crow, MD Bishop, RL Cook, PA Borsa

**Affiliations:** 1 Department of Applied Physiology & Kinesiology, College of Health & Human Performance, University of Florida, Gainesville, FL USA; 2 Department of Orthopaedic Surgery, Georgia Prevention Institute, Augusta University, Augusta, GA USA; 3 Department of Physical Therapy, College of Public Health & Health Professions, University of Florida, Gainesville, FL USA; 4 Department of Epidemiology, College of Public Health & Health Professions, University of Florida, Gainesville, FL USA

**Keywords:** Cannabidiol (CBD), Musculoskeletal, Injury, Pain, Physical disability, Impairment, Safety

## Abstract

**Background:**

Exercise and sports-related musculoskeletal injuries often result in pain, and the recovery process can be prolonged and challenging. Many physically-active Americans have begun using cannabidiol (CBD) for pain relief and improved recovery time, and CBD is of particular interest due to its positive safety profile, non-intoxicating effects, and therapeutic potential. Our objectives were threefold. First, to confirm the safety and tolerability of ingesting a CBD-rich hemp-extract. Second, we examined the feasibility and acceptability of our recruitment and blinding methods, and the tests and measures used to determine preliminary efficacy. Last, we planned to examine within-group effect sizes and observed power to make preliminary determinations of efficacy.

**Methods:**

A randomized, placebo-controlled, double-blind, two-arm parallel study design was used with participants randomly allocated to either a CBD or a placebo control group. Participants completed a 15-day study trial which included baseline testing, intervention (CBD/placebo dosing), and follow-up assessments on days 11 through day 15 (24 to 96-hrs post-injury). Outcomes included pain intensity, strength loss, and physical disability. Participants self-administered the investigational product (CBD or placebo) under the tongue twice daily for 15 days. Daily dosage of CBD was 67 mg. An experimental injury protocol for the quadriceps muscle group on day 10 induced pain and functional disability in otherwise healthy men (*n* = 9) and women (*n* = 20) [age: 20.1 yrs, weight: 64.2 ± 8.5 kg, height: 169 ± 5.3 cm].

**Results:**

There were no reports from participants of side effects or treatment-emergent adverse reactions from consuming the investigational products. Symptoms and functional deficits were most pronounced 24 to 48 h. post-injury and were least pronounced by 96-hrs post-injury. CBD users reported less peak pain at rest and with movement at 48-hrs post-injury. The CBD group showed less strength impairment and physical disability than placebo at 48-hrs post-injury.

**Conclusions:**

Sublingually administered CBD demonstrated a favorable safety profile and showed promise in reducing pain-related symptoms associated with exercise-induced muscle injury. Clinical studies using larger sample sizes are needed to confirm the reported estimates of efficacy in reducing pain and improving function post-injury.

**Clinical trial number:**

NCT04586712, registered on October 14, 2020.

## Introduction

Musculoskeletal injuries can occur accidentally from a trip or fall or can be exercise-induced. Sports participation accounts for over 65% of musculoskeletal injuries and 50% of visits to the emergency room (Patel et al. 2017). Acute inflammatory pain, physical disability and mental anguish are common manifestations. Injury recovery can be time-consuming and difficult for those who avoid seeking medical care due to work obligations or lack of access to health care services. As a result, many people will seek pharmacologic aid for their injuries to help alleviate their pain and facilitate functional recovery. Over the counter (OTC) non-steroidal anti-inflammatory drugs (NSAIDs) or physician prescribed opioid medications are commonly used to provide symptomatic relief. However, prolonged use of NSAIDs and/or opioid medications poses a significant health risk coupled with the rise in dependence for opioid medications (Li et al. 2013). As a result, many physically-active Americans, including prominent professional athletes, have begun using CBD-related products for relief from their bodily ailments and lingering pain (Frane et al. 2022; Docter et al. 2020). The primary medical reason people report using CBD is for pain relief (62%) (Corroon and Phillips 2018), and CBD is viewed as a safe alternative to NSAIDs and opioid-based medications (Reiman et al. 2017; Nguyen et al. 2023). The increase in CBD use reflects a growing interest in alternative treatments for symptom management among active individuals (Rojas-Valverde 2021). Anecdotal reports of CBD use have been positive, with many individuals’ claiming better sleep quality and quicker recovery from muscle and joint soreness and stiffness (Moltke and Hindocha 2021).

Research has shown evidence that phytocannabinoids may have a promising therapeutic potential in a variety of physical ailments, and cannabidiol (CBD) is of particular interest due to its positive safety profile, low abuse potential, non-intoxicating effects, and widespread medicinal capabilities (Maroon and Bost 2018; Corroon and Felice 2019, Alaia et al. 2022; Cochrane-Snyman et al. 2021; Hackett et al. 2020). CBD is one of more than 100 cannabinoids found in the *Cannabis Sativa L.* or hemp plant (El Sohly et al. 2017). Hemp-derived CBD does not produce psychotropic effects unlike other cannabis products such as Δ^8^/Δ^9^-tetrahydrocannabinol (THC) (Li et al. 2021). CBD is chemically similar to components of the endocannabinoid system (ECS) that play an important role in the homeostasis of bodily functions including the regulation of tissue inflammation and pain (Carroon and Felice 2019). CBD can account for up to 95% of the Hemp plant’s extract and does not violate the Controlled Substance Act if it contains less than 0.3% THC by dry weight (Farm Bill 2018) (US H.R. 2018). CBD can be purchased legally on-line or in major drugstores, convenience stores, and medical cannabis dispensaries for human consumption. The sale of CBD related products in the US is poorly regulated even as the hemp industry is rapidly growing. As of 2024, consumer CBD-related sales totaled approximately $9 billion, and is estimated to reach $60 billion by 2030 (Cannabidiol market size 2025, Cheng 2022). The widespread use of CBD has gotten far ahead of the evidence to support the health-related marketing claims being made (Zenone et al. 2021). Therefore, scientific exploration and validation are necessary.

Our primary objectives were threefold. First, to confirm the safety and tolerability of ingesting a CBD-rich hemp-extract. Second, we examined the feasibility and acceptability of our recruitment and blinding methods, and the tests and measures used to determine preliminary efficacy. Last, we planned to examine within-group effect sizes and observed power to make preliminary determinations of efficacy.

## Methods

### Experimental design and study population

This pilot feasibility clinical trial was authorized by the U.S. Food & Drug Administration (IND #147985) and registered with ClinicalTrials.gov (NCT04586712). Institutional Review Board (IRB201903330) approval and participant consent was obtained prior to study enrollment. A double-blind, randomized, placebo-controlled, two-arm parallel study design was used with participants randomly allocated to either an active CBD or a placebo control dose group. Random allocation to treatment group was performed using a computer assisted web-based program. Prior to participant enrollment, a randomization sequence was generated in Microsoft Excel (Microsoft Corp., Redmond, WA, USA) using the *RAND()* function to link participant identification numbers to blinded treatment codes. The randomization list was created by a study team member not involved in data collection, was not accessible to personnel involved in outcome assessment, and was not altered throughout the study. We assessed, in serial fashion, symptomatic response, functional limitations and recovery following induced musculoskeletal injury in which CBD (or placebo) was administered sublingually during a 15-day clinical trial. The 15-day trial consisted of a pre-injury dosing phase (days 0–10), pre-injury baseline measurements and injury induction (day 11) followed by post-injury recovery measurements (days 12–15). 

Recruitment & screening: Participants responded to advertisements posted around UF campus and on course websites. Candidates who responded to the advertisements were contacted by phone for a screening interview to determine if he/she met the entry criteria. 

Inclusion criteria: (a) biological male or female adults between the ages of 18–35 years, (b) English speaking, and (c) both biological female and male subjects agreed to practice acceptable methods of birth control, such as abstinence, or methods of contraception (barriers, oral, patch or other prophylactic methods) during the intervention. 

Exclusion criteria: (a) current use of cannabis products on a regular basis or positive urine test for marijuana (THC), (b) current use of tobacco or nicotine containing products on a regular basis, (c) currently taking prescription medication for management of anxiety disorders, depression, or ADHD, (d) current use of nutritional supplements or OTC anti-inflammatory medications on a daily basis, (e) history of seizure disorder, family history of seizure disorder, current or history of head trauma, liver disease, renal (kidney) disease, cardiovascular disease (including, but not limited to: hypotension, hypertension, tachycardia, and syncope), (f) current medical condition that would prevent the participant from performing strenuous resistance exercise, (g) weight lifting for the lower extremities (legs) more than twice a week, (h) currently experiencing pain in the hips, leg, or knee region, (i) pregnancy, lactating or positive urine pregnancy test, and (j) known allergy to CBD or coconut/sesame oil.

**Fig. 1:**
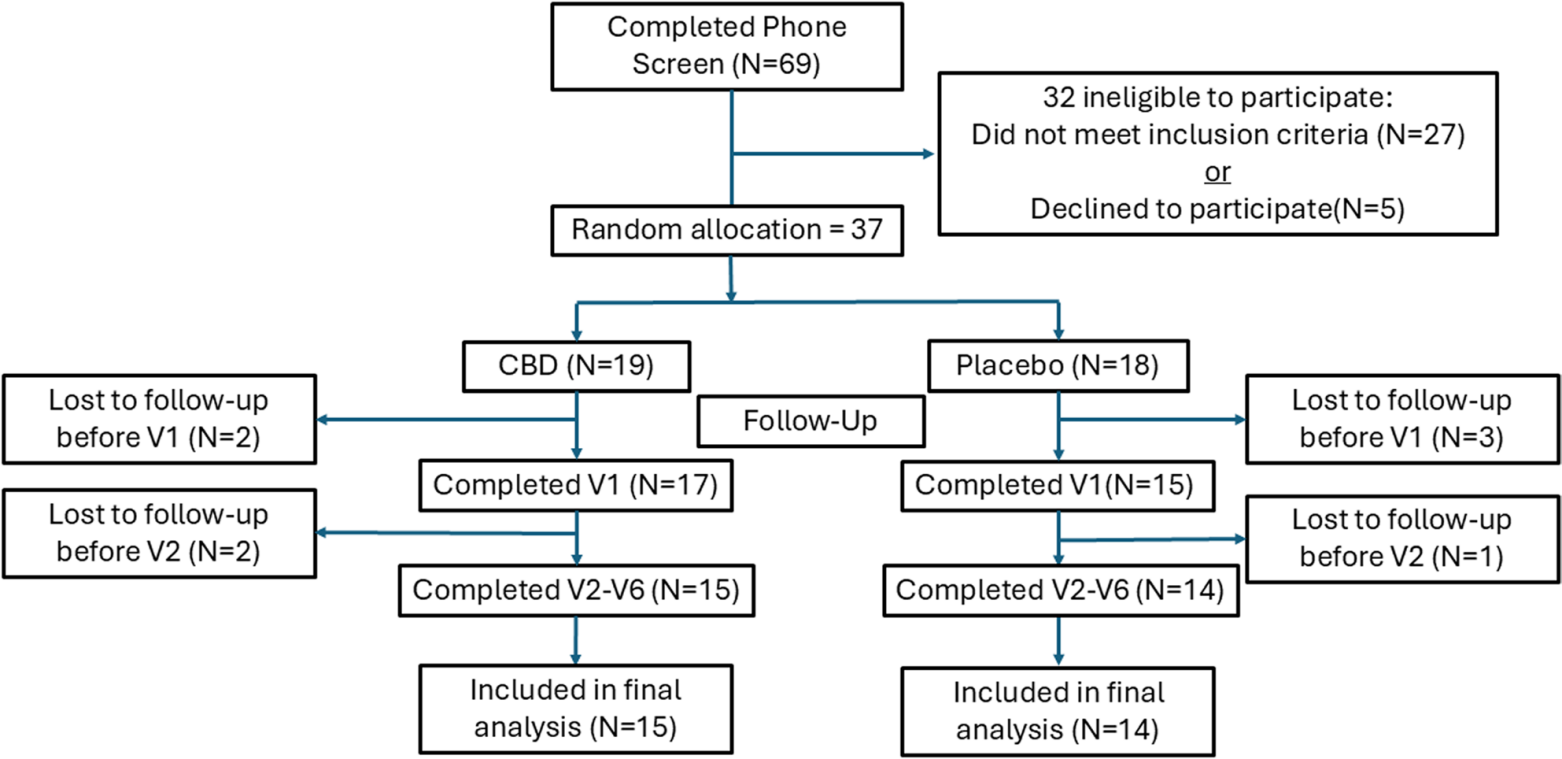
CONSORT Flow diagram

If the candidate was eligible and agreed to participate, he/she was scheduled for their 1st on-site laboratory visit. Participants were required to report to the Sports Medicine Research Laboratory (SMRL) for a total of six scheduled on-site visits. Twenty-nine participants [X̄±SD; age = 20.1 ± 2.1 yrs, height = 169 ± 5.3 cm, weight = 64.2 ± 8.5 kg] completed the study trial (Fig. 1).

#### Study treatment

The investigational products used in the clinical trial included a hemp supplement (CBD) and a placebo control. The hemp-derived CBD was extracted by Socati Montana, LLC (5840 Expressway, Missoula, MT 59808), and shipped to SunFlora, Inc. (411 19th Street South, St. Petersburg, FL 33712) where it was formulated into oil-based tinctures. The investigational product (SunMed™ Hemp Supplement) is a full spectrum CBD-rich formulation containing very low levels of THC (< 0.3%), along with all naturally occurring minor cannabinoids, terpenes, and essential oils from the plant extract. Flavoring agents were added to ensure palatability. The tincture solution was provided in a 30mL bottle (2000 mg CBD) with a syringe dropper. A medium chain triglyceride-based mixing agent blended with coconut oil was used as the placebo-control. The addition of the CBD to the mixing agent did not change the physical properties of the liquid and thus was indistinguishable from the placebo-control. Participants were provided with the tincture on day 1 and instructed to self-administer the solution under the tongue (0.5 cc ~ 1/2 dropper) twice per day (BID) 12 h apart (morning and evening with food) for 15 days. Daily dosage of CBD was 67 mg for the CBD group and 0 mg for the placebo group. 

#### Experimental injury model

The Biodex System 4 Pro isokinetic dynamometer was used for the high intensity resistance exercise (HIRE) protocol. Each participant performed five sets of twenty-five repetitions using concentric and eccentric muscle actions. The angular velocity was set at 60°/sec for concentric and 90°/sec for eccentric muscle actions. Participants were given a three-minute rest period between sets. Verbal encouragement was provided by research technician, and the participant was instructed to perform each repetition with maximal effort.

This particular experimental model was selected because it allows us to control and standardize the mechanism of injury and allows us to track symptomatic response and physical impairment in a more clinically relevant manner when compared with other experimental pain models that are controllable but of shorter duration (e.g. thermal or pressure stimuli). The bout of HIRE is able to systematically optimize eccentric overload of the muscle coupled with fatigue to produce controlled strain-induced damage to the contractile elements of the muscle. The resultant muscle injury produces a local inflammatory response with associated soreness, stiffness, disability, and functional deficits (Lewis et al. 2012). We have also demonstrated an established link between this pre-clinical experimental pain model and a post-operative clinical pain model suggesting robust validity (George et al. 2015, 2017).

### Measures

#### Safety

The occurrence and frequency of adverse reactions to the investigational product was used to track safety and tolerability of the investigational products. Each participant was provided a detailed safety monitoring checklist of side effects and/or adverse reactions that they may potentially experience during the dosing phase of the study. Participants were then instructed on what to do if they experience a side effect or adverse reaction between study visits. Participants were also assessed actively at each on-site visit by study personnel using the side effects/adverse events checklist. Each participant was read each side effect/adverse reaction from the checklist, and the participant was instructed to answer either yes or no.

#### Recruitment and retention

A research assistant recorded the number of candidates who contacted the SMRL to request information about participating in the study. Similarly, attendance at on-site laboratory visits and participants who completed the study were tracked and recorded. 

#### Peak pain intensity

A self-report visual analog scale (VAS) was used to assess the peak intensity of muscle pain experienced by the participants pre-exercise (baseline) and follow-up (24-, 48-, 72- and 96-hours post-exercise). The VAS consisted of a 100 mm horizontal line with 0 (no pain) on the left pole and 100 (worst pain imaginable) on the right pole and participants were asked to rate their level of pain by placing a slash on the line that best represented their worst level of pain over the past 24 h. The score was recorded as peak pain intensity (PPI). A stair climbing test was used to assess movement-evoked pain (MEP) (Ronai and Gallo 2020). Participants were asked to ascend and descend a flight of 10 stairs, and upon completion of the stair climb test the participant was asked to rate their level of perceived pain experienced while performing the stair climbing test using the VAS. 

#### Strength impairment

Quadriceps strength measurements in the form of maximal voluntary isometric contractions (MVICs) were performed before and after completing the experimental injury protocol. MVICs were measured using the Biodex System 4 Pro (Biodex Medical Systems, Inc., Shirley, NY) isokinetic dynamometer. Participants were seated with their dominant leg placed in 60° knee flexion (hip resting in 15° flexion). Each participant performed three knee extension MVICs held for 5 s (30 s rest between contractions), and the maximum score of the three was recorded as peak torque (Newton-meters per Kilogram of body weight or N-m/Kg BW). Verbal encouragement was provided by research technician, and the participant was instructed to perform each repetition with maximal effort. Strength impairment was calculated as the reduction in peak torque from pre-injury to 48-hours post-injury. 

#### Peak disability

Physical disability was measured using Lower Extremity Functional Scale (LEFS) (Binkley et al. 1999). The LEFS is a self-report questionnaire and contains 20 items concerning an individual’s ability to perform everyday tasks. The scale can be used to evaluate the functional impairment of a participant post-exercise. Each item includes a 0–4-point hierarchical grading scale with 0 being “extreme difficulty or unable to perform activity” and 4 being “No difficulty”. Participants rated their level of function for each of the 20 items for a total of 80 points. Each participant’s score was divided by 80 and recorded as a percentage value. LEFS scores were recorded pre- and post-injury and the highest score during this period was recorded as the peak level of disability. 

#### Psychological distress

Psychological distress was measured using the Pain Anxiety Symptom Scale (PASS-20) and Pain Catastrophizing Scale (PCS). The PASS-20 is a 20 item, 5-point rating scale that assesses 4 theoretically distinct components of pain-related anxiety including cognitive anxiety, fear of pain, escape/avoidance behavior, and physiological anxiety (Abrams et al. 2007). The PCS is a 13-item, 5-point rating scale used to assess different thoughts that may be associated with experiencing pain (Osman et al. 1997).

#### Time To Recovery (TTR)

Symptomatic resolution was used as an indicator of recovery after injury induction. TTR was determined when PPI ratings returned to within 5% of baseline values, expressed in days (hours) since injury induction.

#### Study blinding

Participants completed a short survey at the completion of the study asking them to judge whether they had been assigned to the treatment (CBD) or placebo group. 

#### Experimental procedures 

**Fig. 2:**
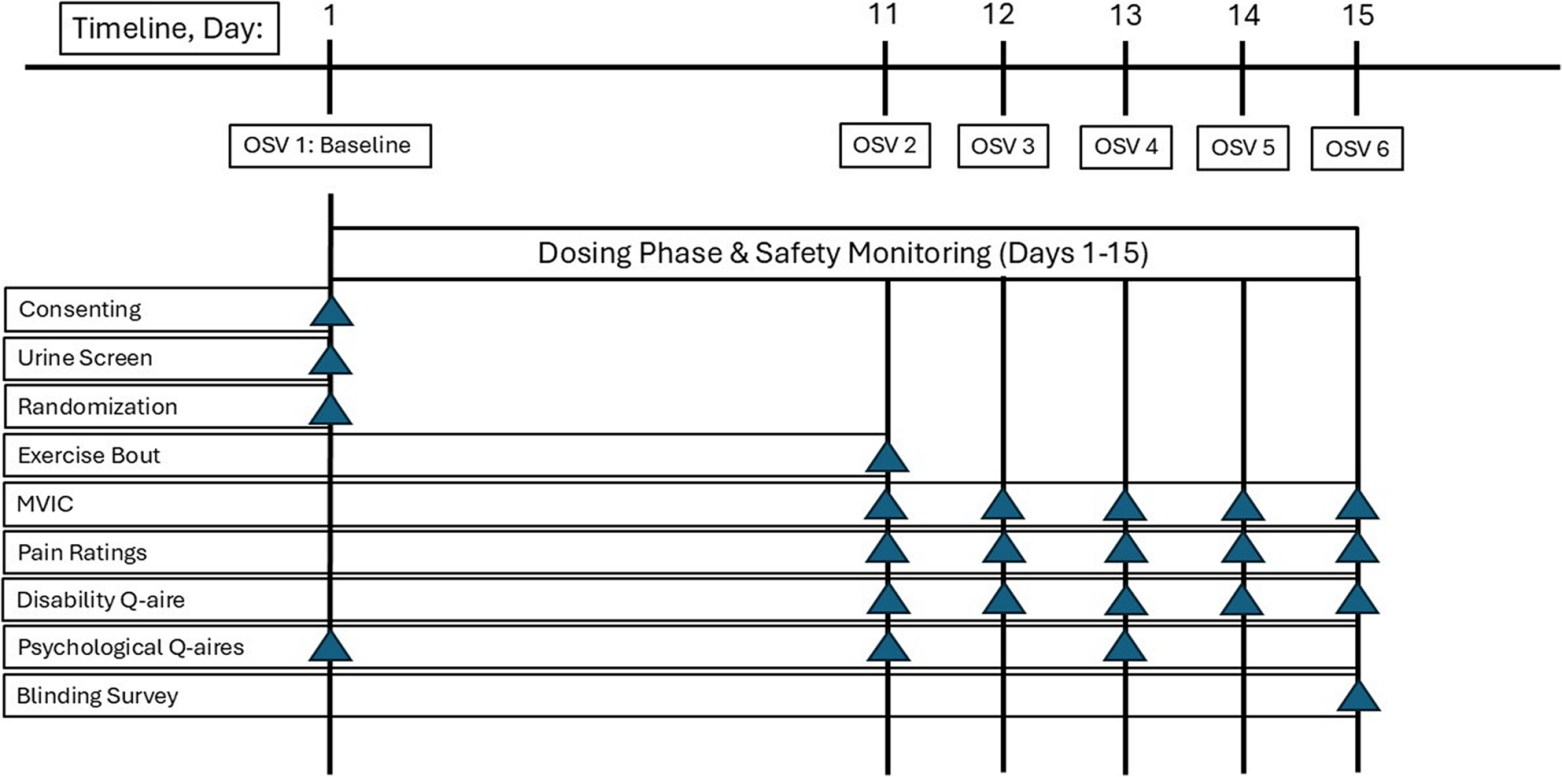
Experimental Procedures. OSV – on-site visit; MVIC – maximal voluntary isometric contraction

During the first on-site visit (OSV) to the lab (day 1), participants were consented and completed self-report psychological questionnaires prior to any testing. Participants provided a urine sample for pregnancy (females) and toxicology screening (presence of cannabinoids), followed by a brief physical exam (including vital signs: measurements for height, weight, pulse, blood pressure and body temperature). Each participant was provided a detailed safety monitoring checklist of side effects and/or adverse reactions that they may potentially experience from ingesting the investigational product. Participants were randomly assigned to a group (CBD or placebo) and instructed on how to administer the investigational product including take-home dosing instructions. Random allocation to treatment group was performed using a computer assisted web-based program on a 1:1 basis (CBD/placebo or placebo/CBD) by study personnel not directly involved in data collection procedures. All participants, investigators, and study personnel will be blinded to treatment order assignments throughout the study. During the second OSV (day 11), participants completed a series of self-report questionnaires (pain, disability, psychological) followed by completion of a leg strength test (MVIC) and the standardized HIRE protocol for the dominant quadriceps muscle group to induce muscle injury. During OSVs 3, 4, 5 and 6 (days 12–15) the same procedures were performed as with the second OSV without the HIRE protocol being performed (Fig. 2).

#### Data management

Data was collected and managed using Research Electronic Data Capture (REDCap) electronic data capture tools hosted at the University of Florida (Harris et al. 2009). REDCap is a secure, web-based software platform designed to support data capture for research studies, providing (1) an intuitive interface for validated data capture; (2) audit trails for tracking data manipulation and export procedures; (3) automated export procedures for seamless data downloads to common statistical packages; and (4) procedures for data integration and interoperability with external sources. 

#### Data analysis 

All data analyses were performed using SPSS^→^ for Windows 16.0 (SPSS, Inc., Chicago, IL). Data were summarized using descriptive statistics (means, standard deviation/error, and confidence intervals), as indicated. One-way ANOVAs were used to calculate effect sizes (partial eta squared or η^2^) and estimates of precision for peak pain intensity, peak physical disability, strength loss, and psychological distress outcomes. Between-group effect sizes were interpreted as small (η^2^ = 0.01), medium (η^2^ = 0.06), and large (η^2^ = 0.14). Survival analysis was performed with a Cox regression and Kaplan-Meier method to estimate the time to recovery (TTR) for peak pain intensity ratings. In this analysis, the censored event was recovery, defined as the timepoint when worst or peak pain intensity rating returned to within 5% of baseline value.

#### Safety

A participant report of experiencing a side effect or adverse reaction during safety monitoring was captured and recorded.

#### Recruitment and retention

The rate of recruitment and retention from initial contact was expressed as a percentage. Completion of on-site visits were calculated for each group and expressed as a percentage.

#### Blinding

A Chi Square test of independence was used to measure the association between the product ingested (CBD, placebo) and the participant’s judgement of which product they were provided to ingest.

#### Efficacy

For each variable, descriptive statistics (means, standard deviation/error, and confidence intervals) and between-group effect sizes (partial eta squared or η^2^) and observed power (1-β) were calculated and compared between groups (CBD/placebo) (Table 1).

**Table 1:** Estimated marginal means, standard deviation/error, 95% confidence intervals, effect size, and observed power

Outcome Measure	N	Mean	Std. D	Std. E	95% Confidence Interval		η^2^	1-β
					Lower Bound	Upper Bound		
Peak Pain (Resting)								
CBD	15	47.8	27.2	7.0	32.7	62.9	0.07	0.29
PLA	14	61.6	22.7	6.3	47.9	75.4		
Movement-evoked Pain								
CBD	15	23.1	21.0	5.4	11.4	34.7	0.10	0.37
PLA	14	37.5	24.3	6.7	22.9	52.2		
Strength Impairment								
CBD	15	24.4	23.6	6.1	11.3	37.5	0.05	0.20
PLA	14	36.7	32.9	8.8	17.7	55.7		
Peak Disability								
CBD	15	67.7	13.2	3.4	60.5	75.0	0.06	0.24
PLA	14	60.1	15.7	4.3	51.2	70.2		

Scores for peak pain and movement-evoked pain were based on a self-reported visual analog scale (VAS) that rated pain from 0 (no pain) to 100 (worst pain imaginable) from pre-injury to 48-hr. post-injury. Scores for strength impairment were based on loss of peak torque (N-m/Kg) from a maximal voluntary isometric contraction (MVIC) of the quadriceps muscle from pre-injury to 48-hr. post-injury. Scores for peak disability were based on a rating scale of 0 (no disability) to 100 (full disability) from pre-injury to 48-hr. post-injury. Std.D - standard deviation; Std.E - standard error; η2 - effect size, 1-β - observed power

## Results

### Recruitment and retention rates

Sixty-nine individuals completed the phone screen to determine if they met entry criteria for the study. Thirty-two (46%) did not meet entry criteria. Thirty-seven (54%) candidates agreed to participate and were randomly allocated to a treatment group (19 CBD, 18 placebo). In both treatment groups four were lost to follow-up between the first and third on-site lab visit. Twenty-nine participants were included in the final analysis (15 in the CBD group, 14 in the placebo group) (Fig. 1).

### Safety and tolerability

No side effects or treatment-emergent adverse reactions from ingesting the CBD or placebo product were reported during the active and passive surveillance periods of safety monitoring.

### Blinding

There was a significant relationship between the two variables [χ^2^_(1,*N*=29)_ = 19.1, *p* < 0.001]. The CBD group was less likely to accurately judge which investigational product they were ingesting during the study. For the CBD group, only 3 out of 15 (20%) accurately judged that they were given the CBD product (12 or 80% thought that they were given the placebo). For the placebo group, all 14 participants (100%) correctly judged that they were given the placebo product.

### Peak pain

The CBD group reported less PPI compared to placebo during periods of rest and less pain intensity evoked with movement at 48-hr. post-injury (Table 1). The data from both pain scores reveal moderate treatment effect sizes.

### Strength impairment

The CBD group demonstrated less strength loss at 48-hr. post-injury compared to placebo (Table 1).

### Peak disability

The CBD group also reported less peak physical disability at 48-hr. post-injury compared to placebo (Table 1).

### Psychological distress

**Fig. 3:**
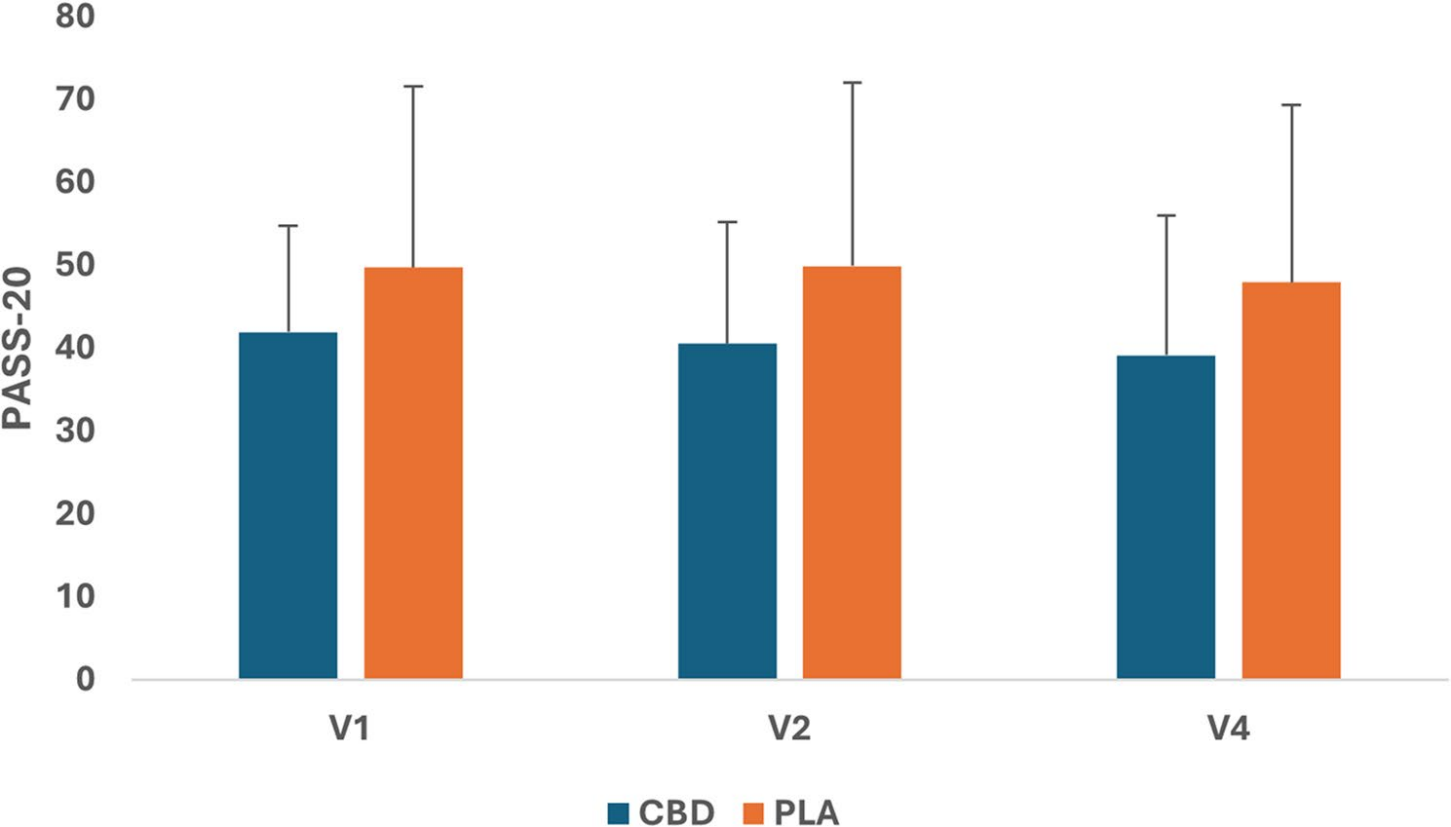
Estimated Marginal Means(± SD) for pain-related anxiety using the Pain Anxiety Symptom Scale-20 (PASS-20). V1 = first visit at baseline (Day 1), V2 = second visit at Day 11 (pre-injury), V4 = fourth visit at follow-up (Day 13 post-injury)

**Fig. 4:**
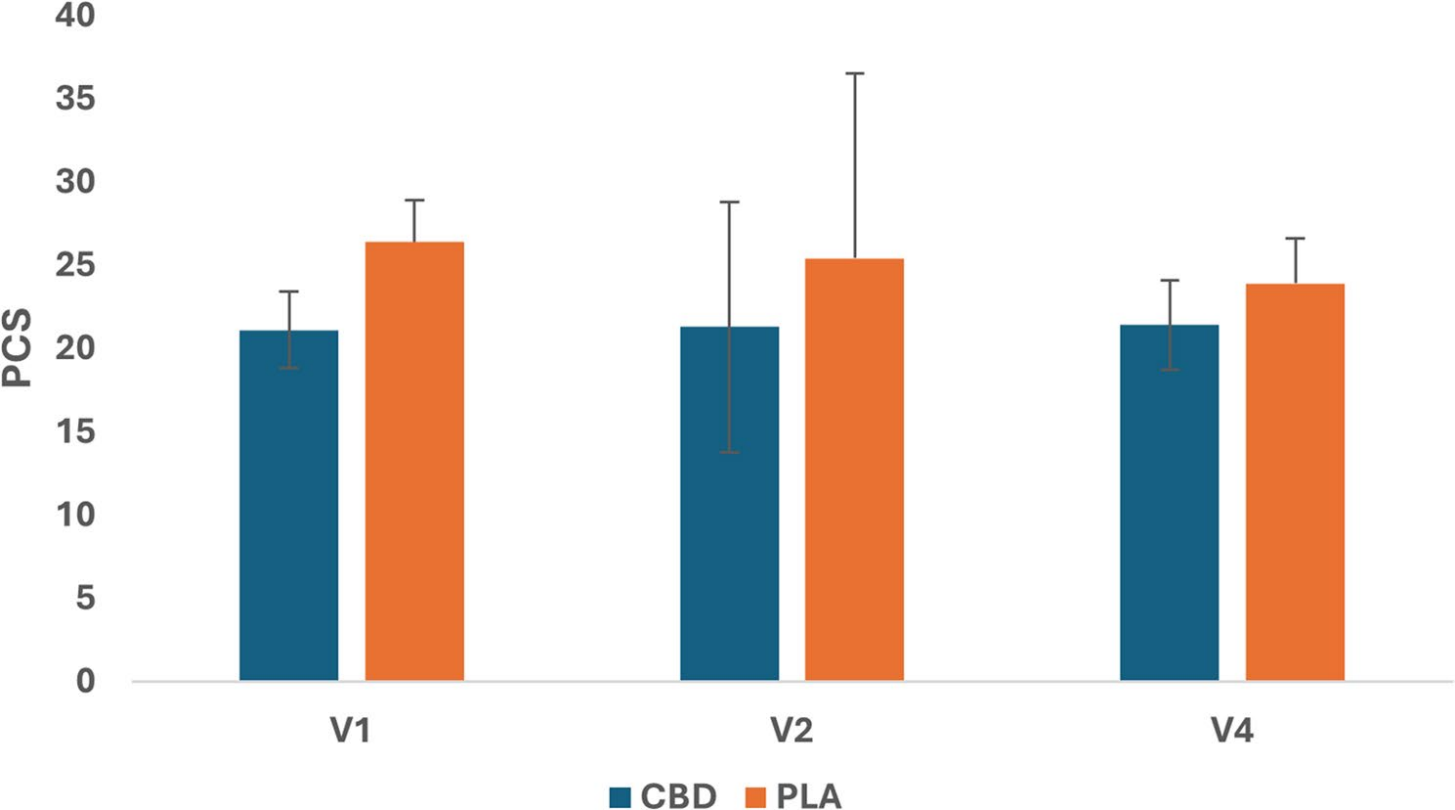
Estimated Marginal Means(± SD) for pain catastrophising using the Pain Catastrophizing Scale (PCS). V1 = first visit at baseline (Day 1), V2 = second visit at Day 11 (pre-injury), V4 = fourth visit at follow-up (Day 13 post-injury)

No significant quantitative changes in psychological distress (pain-related anxiety and catastrophizing) were observed from baseline (day 1) to pre-injury (day 11) and post-injury follow-up on day 13 (48-hr. post-injury) for either group (Figs. 3 and 4).

### Time to Recovery (TTR)

**Fig. 5:**
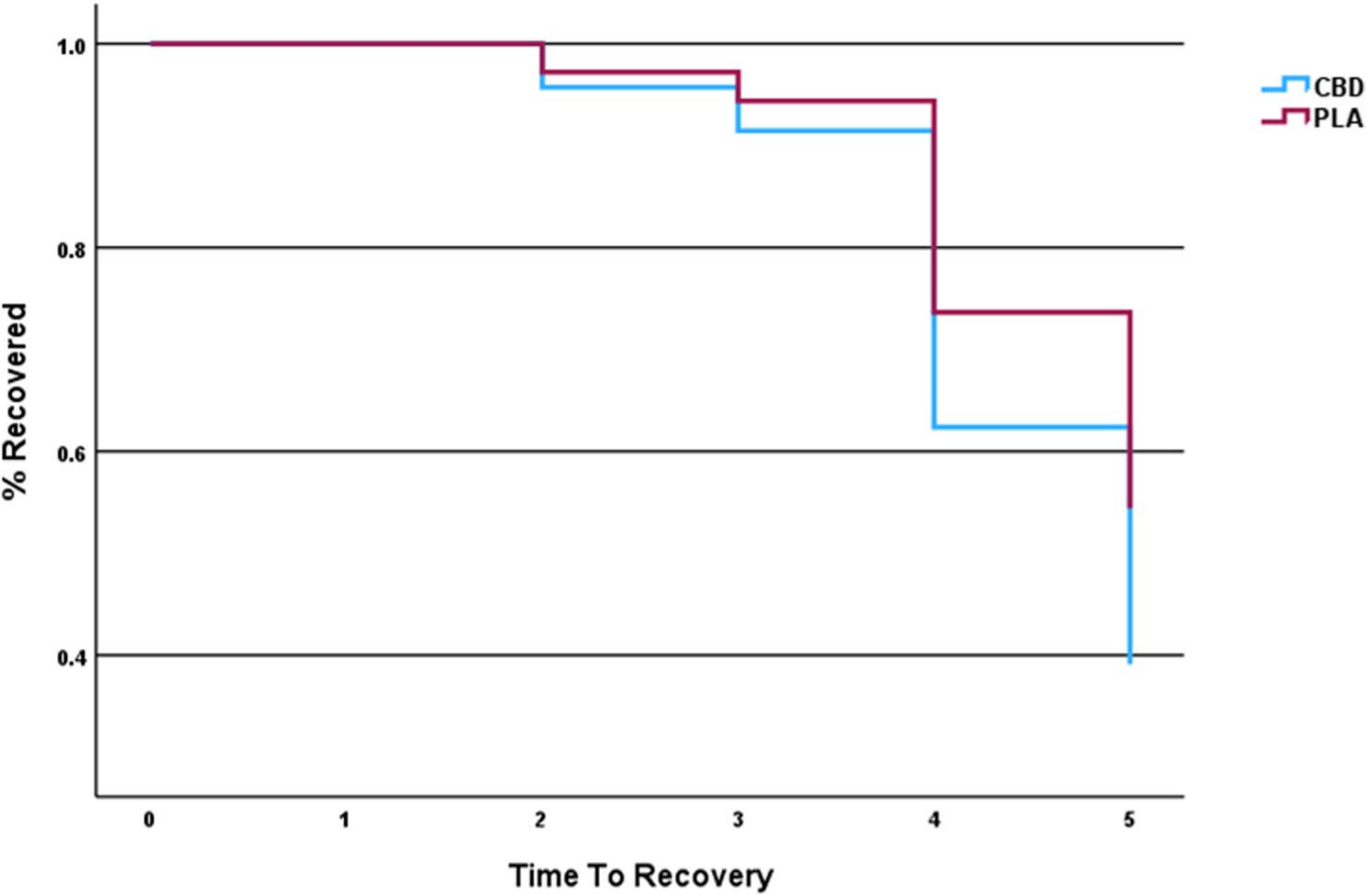
Kaplan-Meier estimates for Time To Recovery (TTR) using peak pain ratings from 0 (Baseline Day 0), 1 (Pre-injury Day 11), and 2–5 (Post-injury Days 12–15)

Survival analysis was performed using a Cox regression analysis with Kaplan-Meier (K-M) estimates. TTR was used as the “event” to evaluate the clinical endpoint for pain resolution. Survival analysis was able to show that our model did not find a significant between-group difference in TTR [B = -0.43; Exp (B) = 0.65; 95% CI 0.23–1.8]. Overall, CBD use did not significantly increase the odds of recoverying faster than taking the placebo (Fig. 5).

## Discussion

Our findings show that the CBD-rich hemp-extract was safe to use as a daily supplement and well tolerated in the short term by participants with no reports of harmful side effects or treatment-emergent adverse reactions. Our acute pain and exercise recovery model was designed to experimentally induce signs and symptoms representative of a strain-induced muscle injury (pain, soreness, stiffness and dysfunction). The muscle fiber disruption and associated connective tissue damage produced a local inflammatory response with symptoms lasting 2 to 5 days after injury induction allowing us to monitor symptomatic and functional recovery. The muscle and connective tissue disruption after exercise induced injury increases the sensitivity of nociceptors and mechanoreceptors within the muscle resulting in discomfort and pain upon muscle activation (MacIntyre et al. 1995).

Our results show medium to large effect sizes favoring lower peak pain at rest as well as pain with movement for participants who used the hemp-derived CBD supplement compared to the placebo control group at 48-hrs post-injury (Table 1). In addition, small to medium effects favoring reduced strength loss and disability for the CBD group compared to placebo were identified suggesting less impairment and quicker functional recovery after injury.

CBD’s exact mechanism of action for modulating pain and inflammation is not presently known; however, several theories have been posited. One theory indicates that CBD works synergistically with the endocannabinoid system (ECS). Elmes et al. (2015) postulated that CBD acts as an endocannabinoid (EC) reuptake inhibitor (like selective serotonin re-uptake inhibitors as an anti-depressant) regulating concentrations of EC ligands, such as *N*-arachidonoyl-ethanolamine also known as anandamide (AEA) and 2-arachidonoylglycerol (2-AG), in the central and peripheral nervous system. The elevated concentrations of these EC molecules are thought to potentiate their intracellular signaling activity in the nervous system, thus exerting anti-nociceptive and anti-inflammatory effects in the body. In vitro and in vivo studies demonstrate that CBD interacts with a variety of G protein-coupled receptors (GPRs), which can protect tissue from secondary damage by downregulating the production of inflammatory cytokines (Ryberg et al. 2007; Laun et al. 2018; Campos et al. 2012; Schuelert and McDougall 2011; Yang et al. 2016). Additionally, CBD’s interaction with CB_2_ receptors is shown to have an inhibitory effect on the COX-2 inflammatory pathway which is directly involved in the development of pain and edema (Pertwee 2008; Ruhaak et al. 2011). CBD has also been found to act as an agonist for transient receptor potential (TRP) ion channels V_1_ and A_1_ located on primary sensory neurons projecting to the spinal cord and brain stem where they modulate pain directly through neuronal desensitization or by interacting with GABAergic inhibitory neurotransmitter circuits (Muller et al. 2018; Bisogno et al. 2001; DePetrocellis et al. 2011).

In clinical studies results were equivocal as to CBD’s efficacy in reducing pain and other pain-related symptoms as a treatment before and after strenuous exercise or arthroscopic surgery. Hatchett et al. (2020) and Cochrane-Snyman et al. (2021) used an eccentric exercise model similar to the one used in the present study to induce muscle damage to the elbow and knee flexor muscle groups. Participants ingested either a CBD or placebo supplement after completing the exercise protocol as a treatment for protecting the muscles from inflammatory damage associated with the strenuous resistance exercise. Hatchett et al. (2020) had participants administer a single dose of CBD or placebo supplement in medium-chain triglyceride (MCT) oil tincture solution (16.7 mg CBD per mL of oil). The CBD group experienced significantly less muscle soreness after exercise compared to the placebo-control group. Additionally, the rate of recovery with CBD use was greater when compared to the placebo-control group. Cochrane-Snyman et al. (2021) had participants ingest CBD or placebo in gelatin capsule form (150 mg per day) for 3 days after completing the exercise protocol. In contrast to the findings of Hatchett et al. (2020), Cochrane-Snyman et al. (2021) did not show a beneficial effect from CBD supplementation compared to the placebo condition for reducing muscle soreness and functional loss. Alaia et al. (2022) conducted a randomized, controlled clinical trial in 100 patients undergoing arthroscopic shoulder surgery. CBD was administered orally in a tablet formulation (75 or 150 mg/day for 14 days) and buccally absorbed. The CBD dose group showed significant reductions in acute pain and higher patient satisfaction scores in the immediate perioperative period after surgery compared with placebo control.

Improving time to recovery after musculoskeletal injury is a highly reported topic of interest in sports science and athletic healthcare (Trecroci et al. 2020; Brooks et al. 2022; Rojas-Valverde and Fallas-Campos 2023). Using Cox regression survival analysis, we were able to track and estimate symptomatic resolution which we used as an indicator of recovery in a controlled manner for 5 days (96-hrs) after exercise-induced injury (Fig. 3). Based on our recovery model, the greatest estimate of between-group separation in the time to recovery occurred on day 4 (72-hrs) with estimates showing that 38% of those participants that used CBD recovered compared to 25% who used the placebo. On day 5 (96-hrs) our estimates show that 60% of those participants that used CBD recovered compared to 45% of those participants that used the placebo. The average time for full symptomatic recovery was 4.4 days for the CBD group, while the placebo group required 4.8 days to recover after injury. This estimate equates to about a half a day (~ 12-hrs) faster recovery rate for the CBD group compared to the placebo group. Timing is a critical factor especially for competitive athletes who need to return to participation expeditiously to prevent deconditioning and optimize skill acquisition during practice sessions (Rojas-Valverde 2021; Rojas-Valverde and Fallas-Campos 2023). Therefore, a half-day difference in the rate of recovery can be considered clinically or practically significant for this particular cohort.

When asked what product they think they were given, most participants (100% in the placebo group, and 80% in the CBD group) indicated that they were given the placebo rather than the CBD. Retrospectively, we postulated that most participants associated using cannabis (marijuana) with “catching a buzz” or feeling “high”. Since CBD is non-intoxicating and, unlike THC, does not cause a “high”, the participants likely assumed that they were given the placebo. In future clinical trials it may be prudent to inform the participant ahead of time that CBD is not the same as THC and will not produce a euphoric response or “high” when ingested. The addition of this step during the consenting period could help to minimize detection bias on the part of participant by preventing any expectation of experiencing a “high” especially for non-naïve cannabis users.

In the current study, participants were informed during the consenting period, both in writing and verbally, that they will likely experience soreness and stiffness within the exercised muscles after completing the strenuous HIRE protocol. Therefore, we postulated that participants would likely experience some level of heightened psychological distress (anxiety and catastrophizing) during the 10-day dosing period before completing the exercise protocol. Participants’ scores at baseline did not change significantly pre- and post-injury indicating that the participants’ level of anxiety and catastrophizing about experiencing soreness and stiffness associated with intense exercise did not cause any additional psychological distress before completing the HIRE protocol.

This was a proof-of-concept study using an acute pain experimental injury model, and larger scale clinical studies with larger sample sizes are needed to confirm our reported trends and indicators of efficacy with CBD use for providing pain relief and functional improvement post-injury. Preliminary data from this study will help in better designing a future clinical trial aimed at identifying an efficacious dose range of hemp-derived CBD extract, as well as determining the cellular and molecular mechanisms that contribute to symptom resolution and recovery. Our findings may also be helpful for making future improvements for treating a broader range of inflammatory conditions that afflict the musculoskeletal system.

The design of the study to focus on safety and feasibility limits the extent to which we can make definitive statements regarding efficacy of our CBD product to modify an acute pain experience following musculoskeletal injury. There was no blood biomarker data to complement our clinical and functional outcomes. Our dosing regimen could be considered on the lower end of the spectrum compared to other clinical trials using CBD as a therapeutic intervention. Several studies cited in the manuscript used single dose designs for CBD administration at higher doses, while our study design utilized a dosing schedule of 15 days of supplementation (10 days prior to exercise-induced injury and 5 days after). Future studies should attempt to use more consistent dosing schedules for better inter-trial comparisons. There were differences in CBD formulations and routes of administration between clinical trials which also can make comparisons problematic and tenuous.

## Conclusions

CBD administered sublingually demonstrated a favorable safety profile and showed promise in reducing symptoms associated with a musculoskeletal injury. Clinical studies using larger sample sizes and broader dose-ranges are needed to confirm the reported trends in controlling pain and function post-injury.

## Data Availability

The datasets used and/or analyzed during the current study are available from the corresponding author on reasonable request.

## Abbreviations

CBD: Cannabidiol
EC: Endocannabinoid
ECS: Endocannabinoid system
THC: Tetrahydrocannabinol
VAS: Visual analog scale
MEP: Movement-evoked pain
NSAIDs: Non-steroidal anti-inflammatory drugs
IND: Investigational new drug
MVIC: Maximal voluntary isometric contraction
LEFS: Lower extremity functional scale
ADHD: Attention deficient hyperactivity disorder
OTC: Over the counter
PCS: Pain catastrophizing scale
PASS-20: Pain anxiety symptom scale – 20
TTR: Time to recovery
SMRL: Sports medicine research laboratory
HIRE: High intensity resistance exercise
OSV: On-site visit
BID: Twice per day
REDCap: Research Electronic Data Capture
K-M: Kaplan-Meier
ANOVA: Analysis of variance
AEA: Anandamide
2-AG: 2-Arachidonylglycerol
GPRs: G protein-coupled receptors
CB: Cannabinoid
TRP: Transient receptor potential
GABA: Gamma aminobutyric acid
MCT: Medium-chain triglyceride
Mg: Milligram
mL: Milliliter
COX: Cyclo-oxygenase
Cm: Centimeter
Kg: Kilogram
